# Vascular Damage, Thromboinflammation, Plasmablast Activation, T-Cell Dysregulation and Pathological Histiocytic Response in Pulmonary Draining Lymph Nodes of COVID-19

**DOI:** 10.3389/fimmu.2021.763098

**Published:** 2021-12-13

**Authors:** Jasmin D. Haslbauer, Carl Zinner, Anna K. Stalder, Jan Schneeberger, Thomas Menter, Stefano Bassetti, Kirsten D. Mertz, Philip Went, Matthias S. Matter, Alexandar Tzankov

**Affiliations:** ^1^ Pathology, Institute of Medical Genetics and Pathology, University Hospital Basel, University of Basel, Basel, Switzerland; ^2^ Department of Biomedicine, University of Basel, Basel, Switzerland; ^3^ Department of Internal Medicine, University Hospital Basel, University of Basel, Basel, Switzerland; ^4^ Pathology, Cantonal Hospital Baselland, Liestal, Switzerland; ^5^ Pathology, Cantonal Hospital Graubünden, Chur, Switzerland

**Keywords:** COVID-19, immunopathology, lymph nodes, macrophage activation, plasmablasts, thrombosis, thromboinflammation, T-cell dysregulation

## Abstract

Although initial immunophenotypical studies on peripheral blood and bronchoalveolar lavage samples have provided a glimpse into the immunopathology of COVID-19, analyses of pulmonary draining lymph nodes are currently scarce. 22 lethal COVID-19 cases and 28 controls were enrolled in this study. Pulmonary draining lymph nodes (mediastinal, tracheal, peribronchial) were collected at autopsy. Control lymph nodes were selected from a range of histomorphological sequelae [unremarkable histology, infectious mononucleosis, follicular hyperplasia, non-SARS related HLH, extrafollicular plasmablast activation, non-SARS related diffuse alveolar damage (DAD), pneumonia]. Samples were mounted on a tissue microarray and underwent immunohistochemical staining for a selection of immunological markers and *in-situ* hybridization for Epstein Barr Virus (EBV) and SARS-CoV-2. Gene expression profiling was performed using the HTG EdgeSeq Immune Response Panel. Characteristic patterns of a dysregulated immune response were detected in COVID-19: 1. An accumulation of extrafollicular plasmablasts with a relative paucity or depletion of germinal centers. 2. Evidence of T-cell dysregulation demonstrated by immunohistochemical paucity of FOXP3+, Tbet+ and LEF1+ positive T-cells and a downregulation of key genes responsible for T-cell crosstalk, maturation and migration as well as a reactivation of herpes viruses in 6 COVID-19 lymph nodes (EBV, HSV). 3. Macrophage activation by a M2-polarized, CD163+ phenotype and increased incidence of hemophagocytic activity. 4. Microvascular dysfunction, evidenced by an upregulation of hemostatic (CD36, PROCR, VWF) and proangiogenic (FLT1, TEK) genes and an increase of fibrin microthrombi and CD105+ microvessels. Taken together, these findings imply widespread dysregulation of both innate and adoptive pathways with concordant microvascular dysfunction in severe COVID-19.

## Introduction

The COVID-19 (Coronavirus Disease 2019) pandemic, caused by SARS-CoV-2 (Severe Acute Respiratory Syndrome Coronavirus 2), has rapidly evolved into the greatest public health crisis of the 21^st^ century. Despite extensive scientific progress in diagnosis, treatment and vaccine development, systematic investigations explicating the immunopathology of COVID-19 are urgently needed, since there is cumulative evidence of its instrumental role in acute disease progression but also in “long COVID” or post-acute sequelae of COVID-19 (PASC) (1–3). While extensive immunophenotyping studies performed on peripheral blood have offered an initial glimpse into the dysregulated host response affecting both innate and adaptive immunity (1, 4, 5), *in-situ* data on the pulmonary draining lymph nodes are currently scarce.

Initial autopsy series have revealed characteristic histomorphology in lymph nodes of severe COVID-19, demarcating it from other viral infections. Predominant features included marked capillary congestion and edema, as well as paracortical increase of plasmablasts, a population of extrafollicular IgG and particularly IgM positive B-blasts, lacking germinal center formation, a predominance of M2-polarized macrophages and hemophagocytic lymphohistiocytosis (HLH) (6, 7). Follicular hyperplasia, the histological correlate of an adequate immune response with immunologic memory, was rarely observed, replaced by an expansion of the abovementioned plasmablasts indicative of a transient humoral response in the absence of germinal center formation (8, 9). Taken together, these features suggest extensive dysregulation in antigen presentation and B-cell activation and response in severe disease, both essential for the development of long-term immunity (10, 11).

Pathological and clinical data have provided ample evidence of an increased incidence of bleeding events and coagulopathies in severe COVID-19 (12–14). In line with these observations, thrombocytopenia was reported to be associated with more severe disease outcomes (15, 16), suggesting that systemic thrombotic microangiopathy plays a central role in COVID-19 pathogenesis. Indeed, a dysregulated interferon gene signature (17–19), resulting in a complex interaction between platelets, endothelium, leucocytes and macrophages, contributes to fibrin deposition, microthrombosis, excess formation of neutrophil extracellular traps (NETosis) (20), cytokine storm (21) and HLH (22, 23). Fibrin microthrombi in multiple capillary beds detected in post-mortem series (7, 12, 24), cytokine-induced complement activation (25), thrombocytopenia with hypersecretion of von Willebrand factor (vWF), angiopoietin 2 and coagulation factor VIII as well as IL-6 and fibrinogen (25–28) all point to a profound dysregulation of the innate immune response leading to thromboinflammation in severe COVID-19. Furthermore, angiotensin converting enzyme 2 (ACE2), the entry receptor for SARS-CoV-2, may play a vital role in platelet activation (29).

An aberrant antigen presentation and resultant disruption of the adaptive immune response is a further immunopathological hallmark of severe COVID-19. Lymphopenia, which has been shown in clinical studies to be negatively associated with disease outcome (30), may be a correlate of systemic T-cell suppression or extensive lymphocyte trafficking into the lungs (31). The abovementioned lack of germinal center formation has been linked to a specific block of BCL6-expressing T-follicular helper cell differentiation, resulting in an accumulation of extrafollicular plasmablasts (9). Due to missing germinal center reaction, these blasts do not undergo somatic hypermutation and class switch recombination, which would typically generate high-affinity antibodies or memory B-cells (8, 32). Instead, incomplete humoral response may result in antibody-dependent enhancement, characterized by the formation of low-affinity, insufficiently neutralizing antibodies. Upon binding to viral antigens, they facilitate direct entry into macrophages (33) or enhance macrophage activation, inducing HLH or Kawasaki-like syndrome as observed in a proportion of patients (23, 34, 35). This process has been previously reported in other viral infections and may be a further pathophysiological principle behind a dysregulation of the adaptive immune response in severe COVID-19, potentially leading to an insubstantial durability of immunity (36). The central role of plasmablasts in COVID-19 has been further substantiated by several immunophenotyping studies of peripheral blood (1, 4, 5, 37, 38).

In order to shed light on the underlying immunopathological mechanisms behind severe disease, we studied the pulmonary draining lymph nodes collected from COVID-19 autopsies by systematic histomorphological, immunohistochemical and transcriptomic analysis in comparison to controls of non-COVID-19 reactive pulmonary lymph nodes.

## Materials and Methods

### Patient Cohort and Study Design

A total of 22 cases of lethal COVID-19 as well as 28 controls were enrolled in this study (**Table 1**). Autopsies of lethal COVID-19 were performed as previously reported (7). Draining lymph nodes were extracted from peribronchial, tracheal and/or mediastinal regions and fixed in 4% buffered formalin for 48 hours before further processing. Control lymph nodes (n=28) from autopsies and biopsy material were selected from a relevant range of histomorphological sequelae, encompassing other infectious processes (infectious mononucleosis lymphadenopathy, n=5, draining lymph nodes of pneumonia, n=1), histopathologic changes reminiscent of COVID-19 [non-SARS related HLH, n=2; mucosa-associated lymph nodes with extrafollicular plasmablast activation n=4; non-SARS related diffuse alveolar damage (DAD) n=3] as well as follicular hyperplasia (n=3) and unremarkable mediastinal lymph nodes, (n=10).This study has received approval by the Ethics Committee of Northwestern and Central Switzerland (ID 2020-00629).

**Table 1:** Clinical and Serological Characteristics of COVID-19 Patients versus Controls.

	COVID-19 (n=22)	Controls (n=28)	p-Value
Age, median years (IQR)	**76 (16)**	**57 (50)**	<0.001**
Sex, male, N (%)	11 (50)	13 (46)	0.513
BMI, median (IQR)	29 (10)	27 (4)	0.336
Hospitalization time, median (days)	9 (12)	9 (9)	0.958
Number of SARS-CoV-2 positive patients, as determined by C_T_ Value (%)	12 (55)	n/a	n/a
Immunosuppressive therapy, N (%)	**2 (9)**	**4 (14)**	0.018*
Steroid therapy, N (%)	**7 (32)**	**5 (18)**	0.041*
High dose dexamethasone (0.4-0.8mg/kg/day)	1 (5)	0 (0)	0.440
Anticoagulation therapy, N (%)	**16 (73)**	**11 (39)**	0.009**
CRP, mg/L, median (IQR)	**191 (182)**	**74 (136)**	0.010**
LDH, U/L, median (IQR)	**444 (441)**	**280 (251.5)**	0.032*
INR, median (IQR)	1.3 (1.1)	1.1 (0.1)	0.150
Thrombocytes, 10^9^/L, median (IQR)	171.0 (291.0)	281.0 (174.0)	0.145
Leucocytes, 10^9^/L, median (IQR)	11.1 (16.6)	9.3 (5.3)	0.773
Neutrophilic granulocytes, 10^9^/L, median (IQR)	7.3 (12.9)	5.0 (10.4)	0.232
Lymphocytes, 10^9^/L, median (IQR)	**0.6 (0.5)**	**1.5 (2.0)**	0.018*
Monocytes, 10^9^/L, median (IQR)	0.4 (0.7)	0.5 (0.6)	0.307

*significant at the 0.05 level, ** significant at the 0.01 level. Bold means significant values.

### Tissue Microarrays, Immunohistochemistry and *In Situ* Hybridization

Representative regions of histological sequelae were annotated by board-certified pathologists on corresponding hematoxylin and eosin (H&E)-stained sections. These regions were transferred to a recipient block using the TMA Grand Master (3DHistech Ltd., Budapest, Hungary), generating three 1.5 mm diameter cores per patient. Immunohistochemistry and *in-situ* hybridization (ISH) for Epstein-Barr virus (EBV) small RNA were then performed on the TMAs using the automated staining system Benchmark XT (Roche/Ventana Medical Systems, Tucson, USA). All primary reagents, retrieval and incubation conditions as well as histological scoring criteria are listed in **Supplementary Table 1**. ISH for SARS-CoV-2 was performed as previously reported (39).

### RT-PCR

To detect SARS-CoV-2, RNA was isolated from the embedded tissues using the Maxwell RSC RNA FFPE Kit (Promega, Madison, WI, USA). A TaqMan reverse transcription polymerase chain reaction (RT-PCR) was performed by using the TaqMan 2019-nCoV Control Kit v1 (ThermoFisher Scientific, Catalog Number A47533) to target three different viral genomic regions (*ORF1AB*, *S* and *N* genes) and the human *RPPH1* gene (RNAse-P). According to the manufacturer’s protocol, a Cт value below 37 in at least two out of three viral genomic regions was considered positive. A case was considered negative if Cт values were above 40. Values between 37 and 40 were considered undetermined and the assay was repeated. Based on the Cт values and quantitation of *RPPH1* transcripts, the viral load was calculated as virus genome copy numbers/10^6^ *RPPH1* transcripts. Samples were always run in duplicates.

### Gene Expression Profiling (GEP)

GEP was performed by HTG according to established protocols (https://www.htgmolecular.com/assets/htg/resources/BR-05-HTG-EdgeSeq-System.pdf). Lysates from samples were run on the HTG EdgeSeq Processor (HTG Molecular Diagnostics, Tucson, AZ, USA) using the HTG EdgeSeq Immune Response Panel with an excess of nuclease protection probes (NPPs) complimentary to their target. S1 nuclease then removed un-hybridized probes, and RNAs leaving behind NPPs hybridized to their targets in a 1-to-1 ratio. Samples were individually barcoded using a 16-cycle PCR reaction to add adapters and molecular barcodes, individually purified using AMPure XP beads (Beckman Coulter, Brea, CA, USA) and quantitated using a KAPA Library Quantification kit (KAPA Biosystems, Wilmington, MA, USA). Libraries were sequenced on the Illumina SEQUENCER platform (Illumina, San Diego, CA, USA) for quantification. Quality control, standardization and normalization were performed by HTG and provided to the investigators. Quality control criteria as determined by the manufacturer (percentage of overall reads allocated to the positive process control probe per sample <28%, read depth ≥750000, relative standard deviation of reads of each probe within a sample >0.094) were met for all samples.

Data was first analyzed by the HTG online tool (https://reveal.htgmolecular.com/), including analysis of the top 20 up- and downregulated genes, differential expression of gene sets according to functional groups and pathways and a manual analysis of the corresponding genes of immunohistochemical markers. A principal component analysis (PCA) was then performed using the pcomp function in R^©^, version 4.0.3 (R-Project for Statistical Computing, Vienna, Austria) and differential expression analysis of COVID-19 cases against controls was conducted with the DESeq2 package using default settings. Sex and age were included in the design formula as additional variables. Count estimates were normalized with the median ratio method, and low-quality samples were excluded from analysis. Prior to the heatmap visualization, the normalized counts were further log2-transformed using a robust variance stabilization. The heatmap including patients’ sex, age and logarithmic viral load was produced with the pheatmap package. The column clusters of the samples as well as the row clusters of the significant genes were obtained by hierarchical clustering with complete linkage and a Euclidean distance metric. The Wald test-statistic was utilized and p-values were adjusted for false discoveries. Adjusted p-values <0.05 and | log2 (fold change) | > 1 were considered significant and included in the analysis.

All expression values were pre-ranked by means of gene set enrichment analysis (GSEA) and their positions compared with the KEGG (Kyoto Encyclopedia of Genes and Genomes), Gene Ontology, GSEA c7 immunology and Hallmark databases. Normalized enrichment scores (NES) > 2 or -2 and adjusted p-values <0.05 were used as preselection criteria for enriched pathways.

### Statistical Analysis

Descriptive statistical methods used to analyze immunohistochemical markers and clinical characteristics are described in the ***Supplementary Methods***.

## Results

### Clinical Characteristics

An overview of demographics and clinical history of both COVID-19 patients and controls can be found in **Table 1**.

### Histomorphology, Immunohistochemical Profiling, ISH, RT-PCR

**Figure 1:**
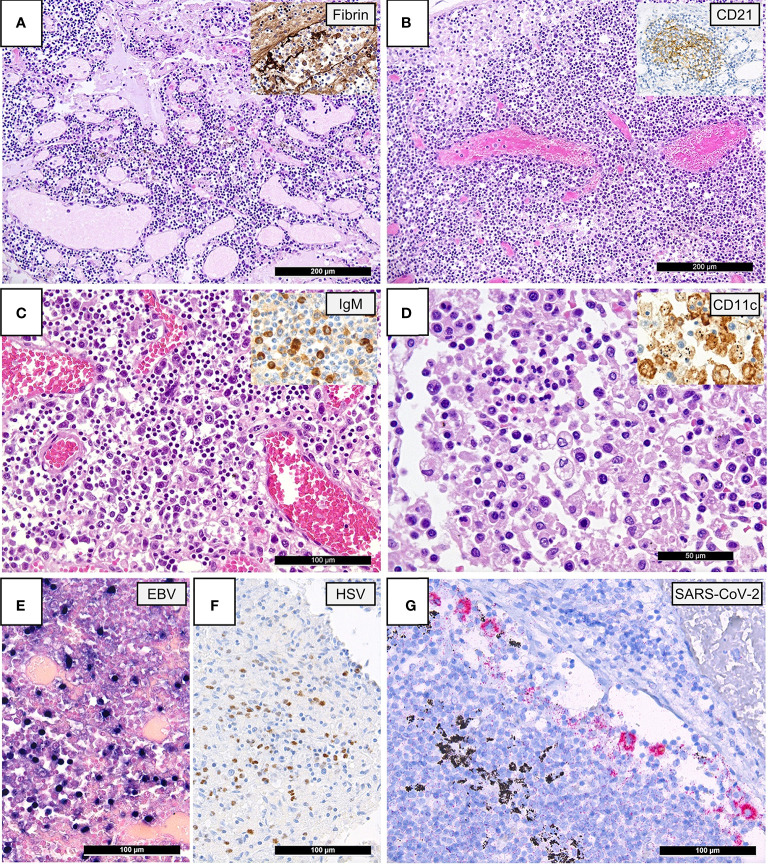
Lymph Node Histomorphology in COVID-19. **(A)** Overview of a lymph node draining a COVID-19 lung with edema and capillary stasis (H&E; 100x); inset: fibrin microthrombus in a dilated subcapsular sinus (immunoperoxidase; 100x). **(B)** Severe capillary stasis and expansion of the paracortex without discernable germinal centers (H&E; 100x); inset: disrupted, CD21+ germinal center network (immunoperoxidase; 100x). **(C)** Proliferation of extrafollicular plasmablasts (H&E; 200x); inset: expression of IgM by plasmablasts (immunoperoxidase; 360x). **(D)** Hemophagocytosis in the sinus of a lymph node (H&E; 360x); inset: positivity for CD11c in histiocytes with hemophagocytosis (immunoperoxidase; 400x). **(E)** Increased amount of EBV-infected B-cells in a lymph node of a COVID-19 patient (EBER ISH; 280x). **(F)** Increased amount of HSV infected B-cells (immunoperoxidase; 280x). **(G)** SARS-CoV-2 positivity in sinus histiocytes as detected by immunohistochemistry for the SARS-CoV-2 N-antigen (immunoperoxidase with 3-amino-9-ethylcarbazole used as a chromogen; 280x).

As previously described in systematic histomorphological analyses on the same autopsy cohort (6), pulmonary draining lymph nodes of lethal COVID-19 displayed the following characteristics compared to controls: 1. vascular changes such as capillary stasis and edema (moderate-extensive 20/21 vs. 5/14; p=0.015) and presence of fibrin microthrombi (6/19 vs. 0/14; p=0.027) (**Figure 1A**); 2. absence or paucity of germinal centers (16/21 vs. 4/14; p=0.013) (**Figure 1B**); 3. increased extrafollicular accumulation of plasmablasts (accumulation in 8/21 vs. 1/14; p=0.05) (**Figure 1C**); 4. an overall increased presence of histiocytes with hemophagocytic activity (moderate-extensive 6/21 vs. 3/14; p=0.037) (**Figure 1D**).

A reactivation of various herpes viruses was observed in a subset of COVID-19 patients by means of immunohistochemistry and ISH [EBV: 4/21 (19%), HSV: 2/21 (9.5%); **Figure 1E**]. The incidence of herpes reactivation was higher in COVID-19 patients than controls [EBV: 2/28 (7%) excluding cases with infectious mononucleosis; HSV: 0/28 (0%), p=0.045]. Importantly, among the 4 COVID-19 cases with EBV reactivation, only one patient was documented as immunocompromised, suffering from therapy-associated myelodysplastic syndrome. VZV was detectable in a lymph node of one non-SARS related DAD, but not in COVID-19. There was no *in-situ* evidence of re-/infection by adenovirus, CMV, HHV8, parvovirus B-19, SV40, nor could SARS-CoV-2 RNA be detected by ISH in the analyzed cohorts. SARS-CoV-2 N-antigen positive macrophages were observed in the sinus of one COVID-19 patient (**Figure 1F**).

**Figure 2:**
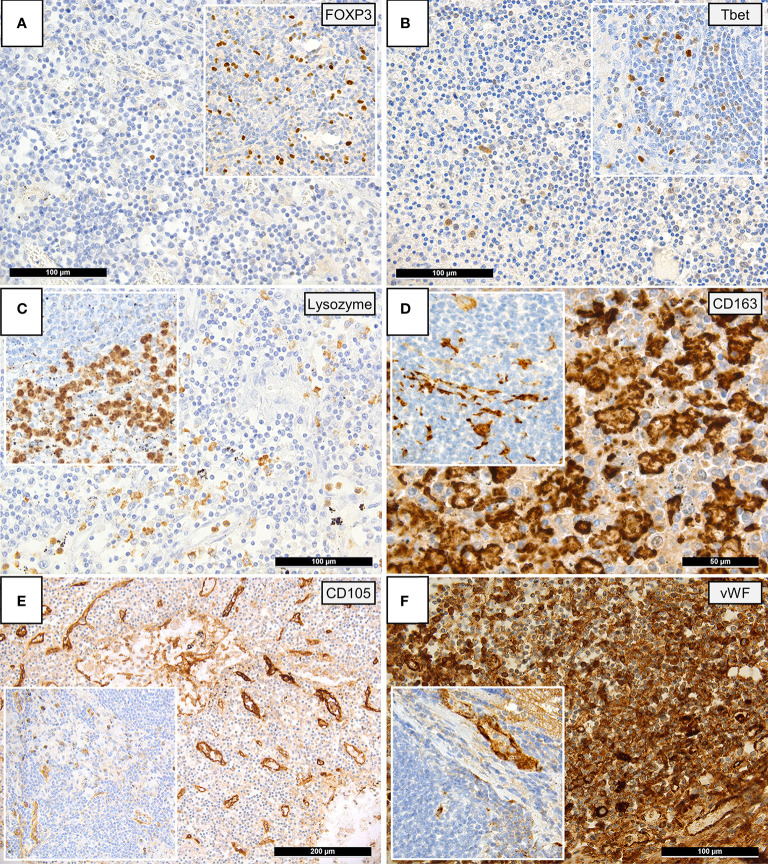
Immunohistochemical Patterns of a Dysregulated Immune Response in COVID-19. Insets display immunohistochemical stainings in controls. **(A)** Paucity of FOXP3-positive Tregs in COVID-19 patients compared to controls (immunoperoxidase; 280x). **(B)** Reduced amounts of Tbet positive T-helper 1 (TH1) cells in COVID-19 versus controls (immunoperoxidase; 280x). **(C)** Reduced amounts of lysozyme-positive histiocytes/macrophages in the paracortex of COVID-19 lymph nodes versus controls (immunoperoxidase; 280x). **(D)** Increased amounts of CD163-positive histiocytes/macrophages in the paracortex of COVID-19 lymph nodes versus controls (immunoperoxidase; 400x). **(E)** Increased CD105-positive microvessel density in COVID-19 lymph nodes versus controls (immunoperoxidase; 50x). **(F)** Increased amount of vWF expression in COVID-19 lymph nodes, vWF positivity in controls is limited to the vascular wall (inset) (immunoperoxidase; 280x).

**Figure 3:**
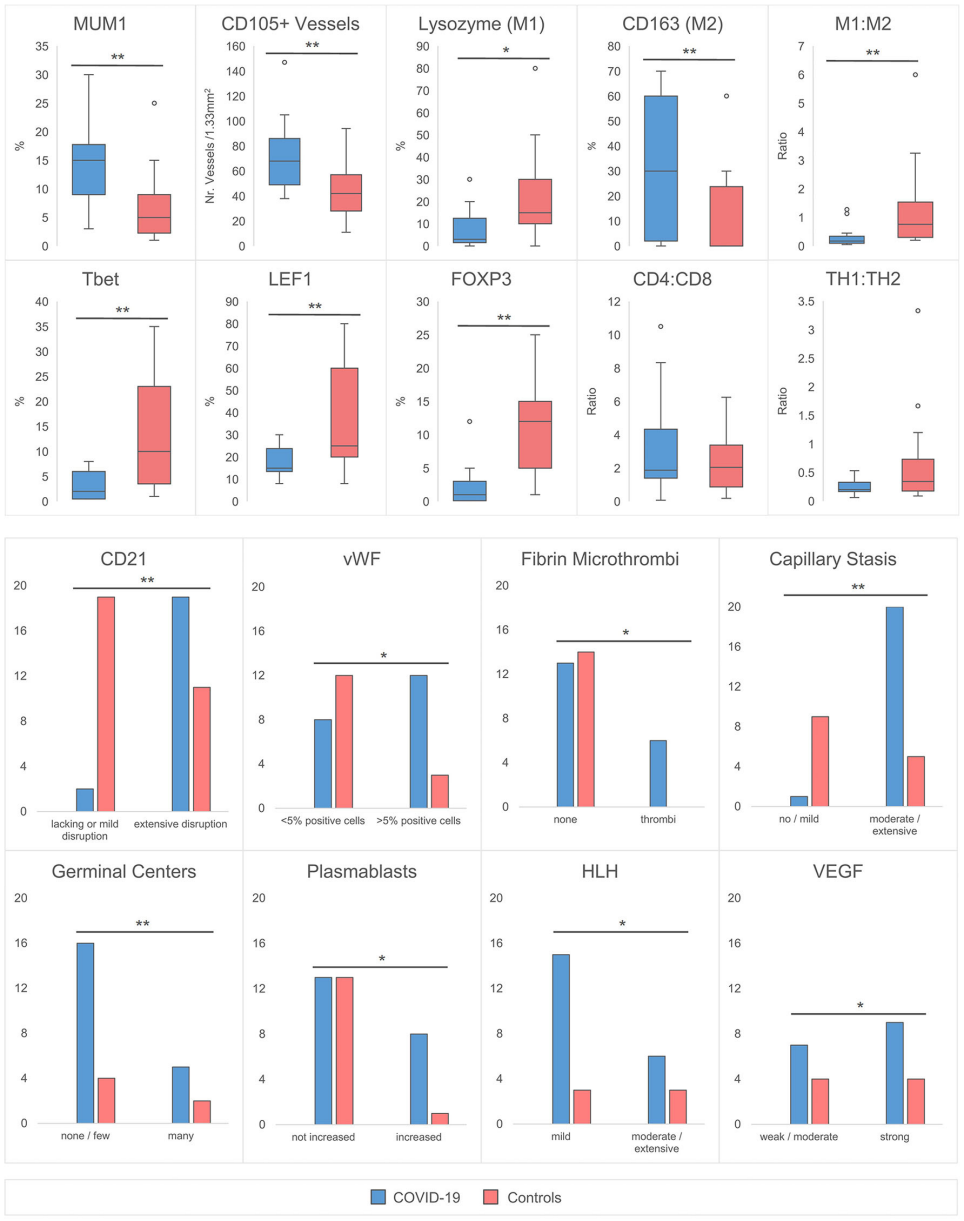
Quantitative Histomorphological and Immunohistochemical Characteristics between COVID-19 Cases and Controls. A significantly higher % of MUM1+ plasmablasts, CD105+ capillaries and CD163+ macrophages is observed in COVID-19. In contrast, the % of lysozyme positive macrophages, Tbet, LEF1 and FOXP3+ T-cells was decreased. The M1:M2 (lysozyme:CD163) ratio is decreased, while CD4:CD8 and TH1:TH2 (Tbet : GATA3) ratios remain unchanged. An increased incidence of CD21+ FDC network disruption with an accompanied lack of germinal centers, increase of plasmablasts, microthrombosis, stasis/edema and moderate/extensive hemophagocytosis is observed in COVID-19 versus controls. The incidence of >5% vWF positive cells and the expression of VEGF is increased. *significant at the 0.05 level, ** significant at the 0.01 level. HLH designates increased hemophagocytic activity of histiocytes, but not hemophagocytic lymphohistiocytosis.

Immunohistochemical findings are shown in **Figure 2**, while histological and immunohistochemical descriptive data are shown in **Figure 3**. Compared to controls, COVID-19 cases displayed a considerably shrunken follicular B-cell compartment (paucity of germinal centers, see above) with a disruption of CD21-positive follicular dendritic cell (FDC) networks (19/21 disrupted in COVID-19 vs. 11/14 in controls; p<0.001) (**Figures 1B** and **3**), while showing a median of 15% of MUM1p-positive plasmablasts compared to 5% in controls (p=0.001) (**Figure 3**); these plasmablasts expressed mainly IgM and IgG, and the amount of IgA-positive plasmablasts and plasma cells was decreased in COVID-19 (data not shown). Major differences applied to the paracortical T-cell zones, to the histiocytic and vascular compartment of the lymph nodes. While the relative amount of T-cells was not decreased in COVID-19, a particular paucity of FOXP3-positive regulatory T-cell-equivalents (Treg) (median 0.75% compared to 12%, p<0.001) (**Figures 2A** and **3**), Tbet-positive TH1-equivalents (median 2% compared to 12%, p=0.001) (**Figures 2B** and **3**) and decreased amounts of LEF1-positive T-cells (median 15% compared to 25%, p=0.009) were observed (**Figure 3**). There were no perceptible immunohistochemical differences of ACE2, CD25, T-cell receptor (TCR)-βF1, TCRδ and RORγt between controls and COVID-19 lymph nodes (data not shown). Additionally, an increased amount of especially sinus-associated CD163 (and CD206)-positive M2-polarized macrophages (median 30% compared to 0%, p=0.011) (**Figures 2D** and **3**) and a decreased amount of both sinus-associated and paracortical lysozyme (and HLA-DR)-positive M1-polarized macrophages was noted in COVID-19 (median 4% compared to 15%, p=0.027) (**Figure 2C**). While CD4:CD8 and TH1:TH2 ratios were not significantly skewed in COVID-19 compared to controls, a significantly lower M1:M2 ratio was observed in COVID-19 [median 0.17 (mean 0.32) in COVID-19 compared to 0.76 (1.34) in controls, p=0.001] (**Figure 3**). COVID-19 lymph nodes contained more CD105-positive (partially newly formed) vessels (median 68/1.33mm² vs. 44/1.33mm², p=0.002), showed significantly higher VEGF expression (median 3 vs. 2, p=0.045) as well as a higher presence of vWF-positive mononuclear cells in the paracortical zones (8/20 cases vs. 3/14 cases with >5% positive cells, p=0.038) (**Figure 3**). Of all above, only CD105-positive vessel density was linked to outcome, with patients dying earlier showing densities over the ROC-determined cut-off score of >67/1.33mm² (**Supplementary Figure 1**). Detailed correlation plots between each immunohistochemical marker and clinical characteristics are provided in **Supplementary Figure 2**.

### Gene Expression Profiling, Pathway Enrichment and Cluster Analysis

**Figure 4:**
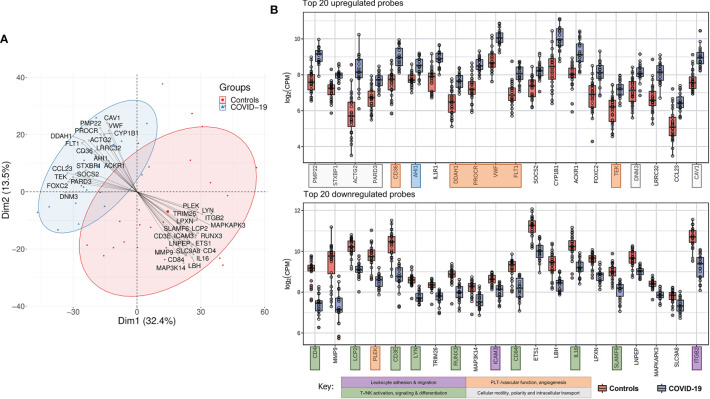
Characteristic Gene Expression Profiling of COVID-19 Lymph Nodes. **(A)** Principal component analysis biplot showing distinct clustering of COVID-19 and controls in whole tissue analysis. Top up- and downregulated genes are superimposed as vectors determining the individual clusters. **(B)** Top 20 up-regulated genes in COVID-19 versus controls: genes related to cellular structure, motility and intracellular transport, proangiogenic and prothrombotic genes were predominantly upregulated in COVID-19. Genes involved in T-/NK- and B-cell differentiation and signaling and leukocyte trafficking were predominantly down-regulated in COVID-19.

A characteristic gene expression pattern in COVID-19 patients compared to controls is demonstrated in **Figure 4**. ***A*** PCA biplot of gene expression profiling between COVID-19 patients and control lymph node tissue is shown in **Figure 4A**. COVID-19 patients distinctly cluster apart from controls with no significant overlap, indicative of a characteristic gene expression profile. This is driven by a group of top up- and downregulated genes (as shown by the collective bundling of arrows in the distinct directions of the clusters). Gene expression profiles in controls were more heterogeneous; cases mapped in proximity to the COVID-19 cluster included one case each of infectious mononucleosis, DAD and draining lymph node of pneumococcus pneumonia. COVID-19 cases in proximity to the control cluster featured cases with longer hospitalization time, notably the one case with HLH (**Figure 1D**) (35).

Top up- and downregulated genes in COVID-19 patients versus controls are shown in **Figure 4B**. Genes encoding for cellular structure, motility and intracellular transport were predominantly found amongst the 20 most upregulated (**genes marked in pale grey**). *PMP22* (peripheral myelin protein 22), *ACTG2* (actin gamma 2, smooth muscle), *PARD3* (par-3 family cell polarity regulator) and *CAV1* (caveolin 1) encode for cytoskeletal and/or membrane proteins responsible for cell motility, division and structure. Essential genes involved in intracellular transport and vesicle formation were similarly upregulated; these include *STXBP1* (syntaxin binding protein 1), *AHI1* (Abelson helper integration site 1) and *DNM3* (dynamin 3). An increased expression of genes associated with vascular function, development and hemostasis likely reflects the previously described microangiopathy in severe COVID-19 (**genes marked in orange**). This is exemplified by a significantly upregulated expression of *CD36*, encoding for platelet glycoprotein 4, and *vWF*, both essential for efficient platelet adhesion and function, a significant downregulation of *PLEK(-strin)*, and by the increased expression of genes involved in angiogenesis and vascular development (*PROCR*, protein C receptor; *FLT1*, Fms related receptor tyrosine kinase 1/vascular endothelial growth factor receptor 2; *TEK*, angiopoeitin 1 receptor). An upregulation of *CYP1B1* (cytochrome P450 family 1 subfamily B member 1) indicates a dysregulation in arachidonic acid metabolism (40). An additionally upregulated gene, *DDAH1* (dimethylarginine dimethylaminohydrolase 1), plays an important role in nitric oxide (NO) synthesis, and implies a state of oxidative stress in chronically inflamed tissue, in line with previous reports of NO/ROS imbalance and decreased NO bioavailability in critically ill COVID-19 patients (41, 42). Furthermore, an upregulation of *IL1R1* (interleukin 1 receptor type 1), *SOCS2* (suppressor of cytokine signaling 2) and *ACKR1* [atypical chemokine receptor 1 (Duffy blood group)] implies a dysregulated cytokine/chemokine response, which may be the correlate of the cytokine storm observed in severe COVID-19 (28).

A severely dysregulated adaptive immune response (**genes marked in green**) is reflected in the downregulation of genes predominantly responsible for T-/NK- and B-cell differentiation and signaling. These include *CD4*, *LCP2* (lymphocyte cytosolic protein 2), *CD3E*, *LYN*, *CD84*, *IL16* and *SLAMF6*. Many of these genes are essential regulative players in leucocyte differentiation and activation, cytokine and integrin signaling and TCR signal transduction. Additionally, a significant downregulation of *ITGB2* (integrin subunit beta 2) and its ligand *ICAM3* (intercellular adhesion molecule 3) implies an inhibition of leukocyte adhesion and migration (**genes marked in purple**), further supported by a significantly lower expression of *MMP9* (matrix metalloprotease 9), an enzyme previously shown to be involved in local proteolysis of the extracellular matrix essential for leucocyte and macrophage movement and chemotaxis (43–45).

**Figure 5:**
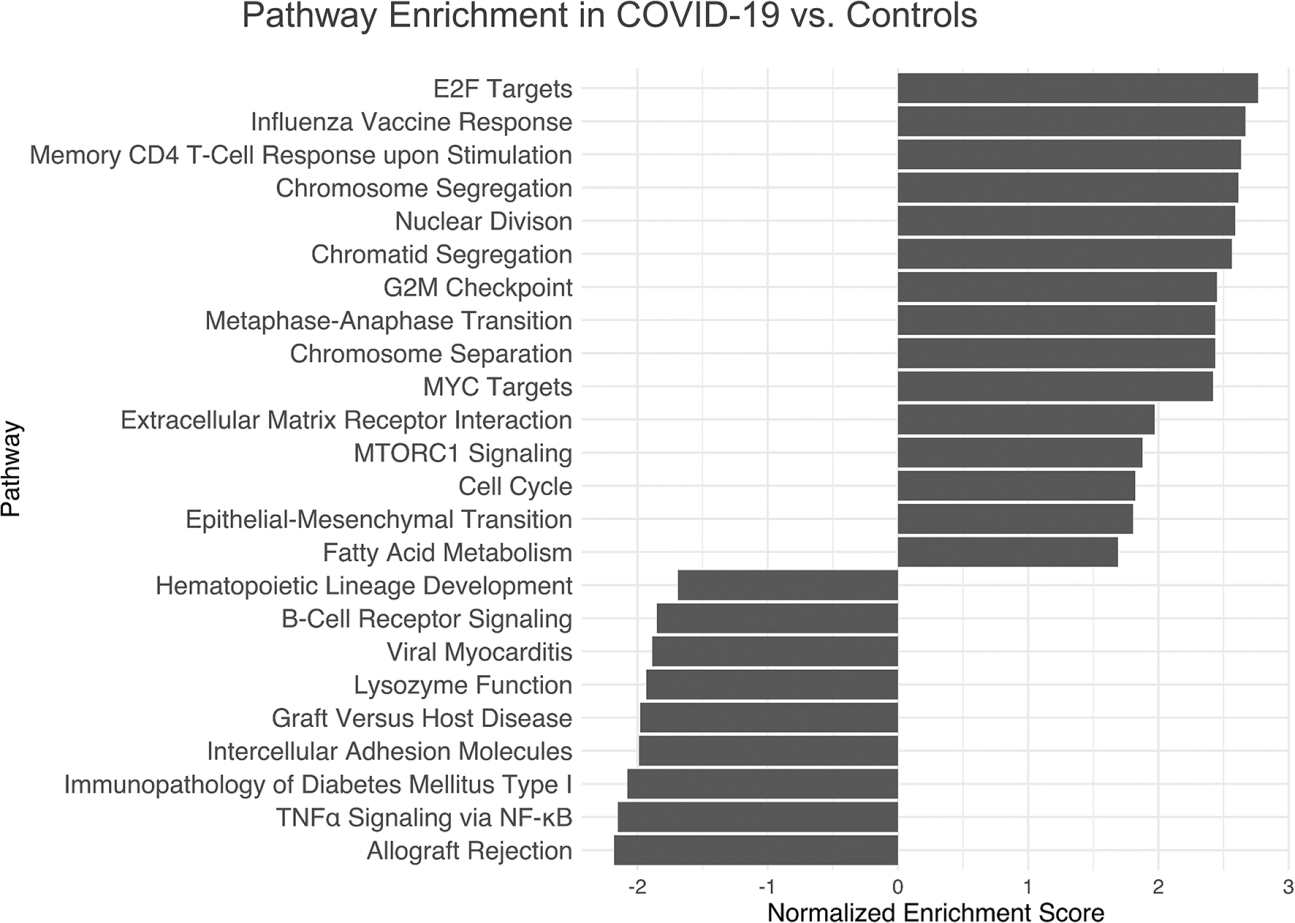
Pathway Enrichment Analysis of Gene Expression Profiling in COVID-19 Immunology. Top up- and downregulated pathways of the GSEA, Hallmark and KEGG database with an abs(NES) > 1. Cell cycle and memory T-cell-response upon influenza vaccine-stimulation, MYC targets and mTORC1-signaling as well as epithelial-mesenchymal transition and fatty acid metabolism related pathways are significantly upregulated in COVID-19, while pathways involving hematopoietic cell lineage development, B-cell receptor signaling, lysosome functions, leucocyte adhesion, TNFα signaling *via* NF-κB and allograft rejection are downregulated. Further details are found in **Supplementary Materials, Table 2**.

Pathway enrichment analysis by means of GSEA, Gene Ontology and KEGG pathway sets revealed cell cycle related pathways, influenza vaccine mediated memory T-cell-response, MYC targets, mTORC1-signaling, epithelial-mesenchymal transition and fatty acid metabolism related pathways as the most significantly upregulated in COVID-19 versus controls. Pathways involving hematopoietic cell lineage development, B-cell receptor signaling, lysosome function, leucocyte adhesion, TNFα signaling *via* NF-κB and allograft rejection were the most downregulated (**Figure 5**). These results correspond to immunohistochemical results (**Figures 2**, **3**). A full list of pathway names and links can be found in **Supplementary Table 3**.

**Figure 6:**
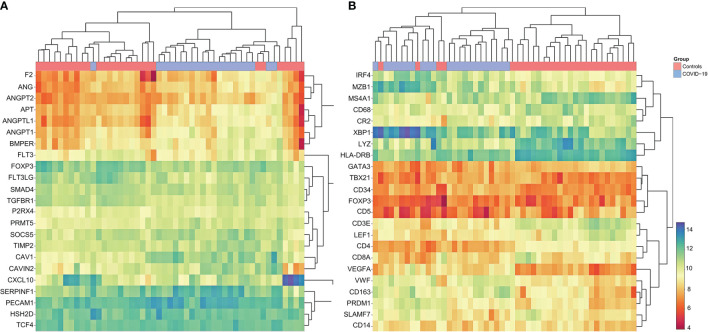
Cluster Analysis of Gene Expression Profiling in COVID-19. **(A)** Marked dysregulation of angiogenesis-related genes in COVID-19 compared to controls; upregulation of ANG, ANGPT2, AGT, ANGPTL1 ANGPT1, BMPER, SERPINF1 and PECAM1; note that COVID-19 cases cluster together, indicating concordance between individual cases. Individual p-values to be found in **Supplementary Materials 2.5**. **(B)** Customized gene set enrichment and clustering analysis of related markers analyzed also by immunohistochemistry shows distinct clustering between COVID-19 and controls: downregulation of CR2 (encodes CD21), TBX21 (encodes Tbet) and LEF1; upregulation of plasmablast genes IRF4, MZB1, XBP1, PRDM1 and SLAMF7; upregulation of angiogenesis related genes (CD34 and VEGF) and of VWF; downregulation of LYZ (encoding for lysozyme) and upregulation of CD163 indicating M2 polarization of macrophages. Note that COVID-19 cases cluster together, indicating concordance between individual cases.

Gene set enrichment and clustering analysis according to the HTG Immune Response Panel revealed a general upregulation of angiogenesis related genes in COVID-19 (**Figure 6A**): these include *ANG* (angiogenin) (fold change 1.52; p<0.01), *ANGPT2* (angiopoietin 2) (fold change 1.61; p<0.01), *AGT* (angiotensinogen) (fold change 2.03; p<0.01), *ANGPTL1* (angiopoietin like 1) (fold change 2.05; p<0.01), *ANGPT1* (angiopoietin 1) (fold change 1.99; p<0.01), *BMPER* (bone morphogenic protein binding endothelial regulator protein) (fold change 1.68; p<0.01), *SERPINF1* (serpin family F member 1, encoding for α2-antiplasmin) (fold change 1.89; p<0.01) and *PECAM1* (platelet and endothelial cell adhesion molecule 1) (fold change 1.25; p=0.05), in line with the vascular dysfunction and hypercoaguable state observed in COVID-19 (12–14). Individual p-values can be found in **Supplementary Table 2**. An upregulation of complement-related genes and a downregulation of T-cell checkpoint related genes was observed (see **Supplementary Figures 3**, **4**), in line with previous observations (17).

In an effort to correlate phenotypic data (**Figures 2**, **3**) with transcriptomics, supervised manual clustering analysis of immunohistochemically analyzed markers was performed (**Figure 6B**). *TBX21* (T-Box transcription factor 1), which encodes Tbet, was downregulated in COVID-19, mirroring the significant decrease of Tbet positive cells as shown in **Figures 2B** and **3**. Analogous observations were made for *FOXP3* and *LEF1* (**Figure 3**). *CR2* (Complement receptor 2) encoding for CD21 was decreased, reflecting the disruption of CD21+ FDC networks (**Figures 1B** and **3**). Genes involved in plasmablast differentiation [*IRF4* (interferon regulatory factor 4), *MZB1* (marginal zone B and B1 cell specific protein), *XBP1* (X-box binding protein 1), *PRDM1* (PR/SET domain 1) and *SLAMF7* (SLAM family member)] were upregulated, reflecting the accumulation of plasmablasts seen histomorphologically (**Figure 1C**). The upregulation of angiogenesis-related genes (*CD34* and *VEGF*) and of *VWF* mirrored immunohistochemical results (**Figures 2E, F** and **3**). Macrophages were M2-polarized, as reflected by a downregulation of *LYZ* (encoding for lysozyme), and an upregulation of *CD163* (mirroring results of **Figures 2C, D** and **3**).

**Figure 7:**
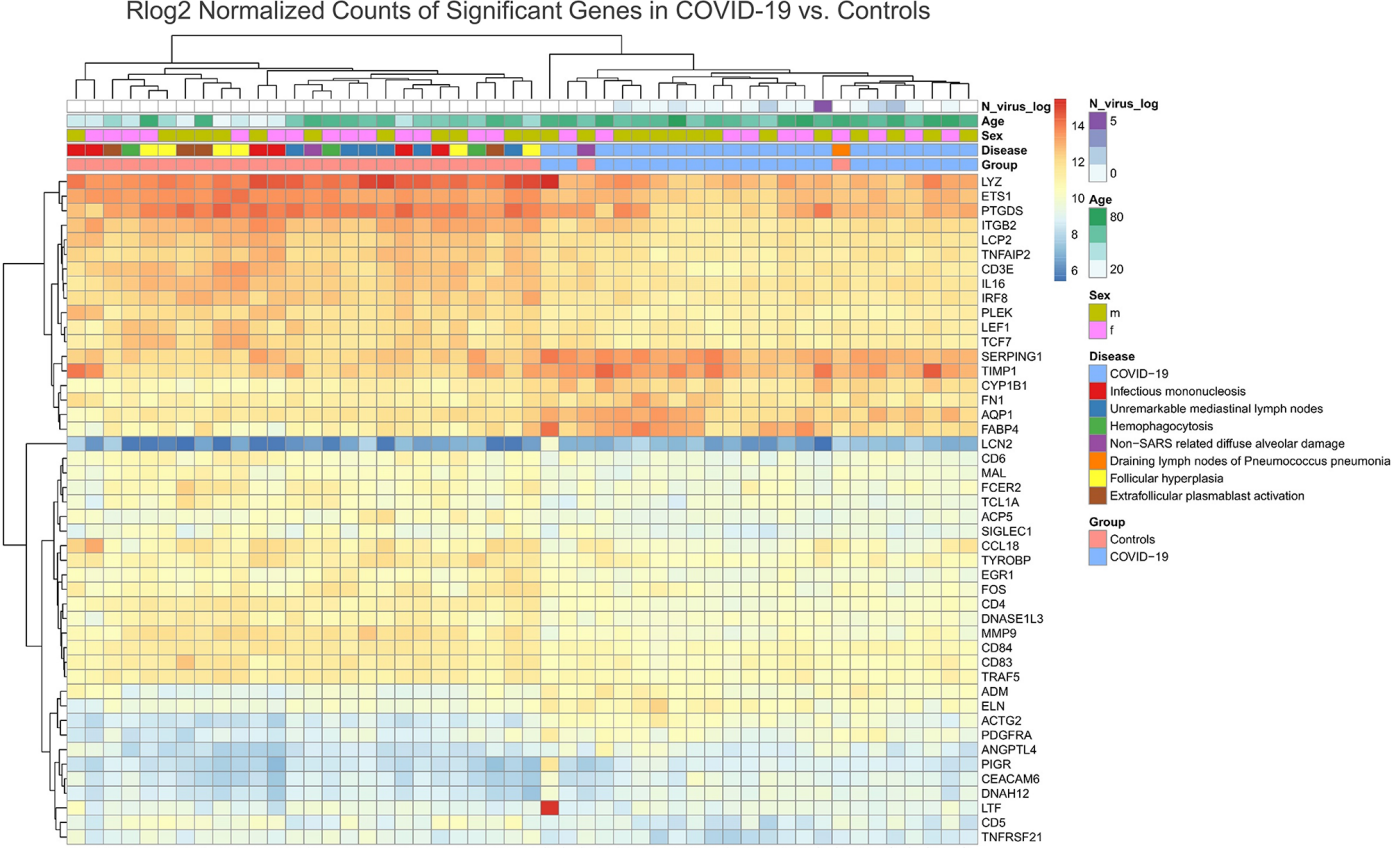
Unsupervised Cluster Analysis of Differentially Expressed Genes in the Draining Lymph Nodes of COVID-19 Compared to Control Cases. A downregulation of genes related to T-cell function and differentiation (LCP2, CD3E, LEF1, CD6, MAL, TCL1A, TYROBP, CD84, CD5), in particular CD4 associated (IL16, CD4) and genes encoding for immunoglobulin receptors (FCER2, PIGR) and transcription factors (ETS1, EGR1, FOS) was observed in COVID-19 patients versus controls. A downregulation of LYZ and SIGLEC1 indicates an altered macrophage phenotype (less prevalent M1 polarization). Genes related to complement and fluid homeostasis and vascular functions (SERPING1, AQP1, ADM, ANGPTL4) are upregulated. A dysregulation in expression amongst genes encoding for leukocyte adhesion and motility was observed (downregulation of IRGB2, upregulation of ACTG2 and DNASH12). MMP9 is downregulated while its inhibitor TIMP1 is upregulated. Other genes involved in remodeling (ELN, PDGFRA and FN1) are upregulated. A downregulation of PTGDS and upregulation of CYP1B1 indicates an altered arachidonic acid metabolism.

Unsupervised cluster analysis revealed a unique differential gene expression of 46 genes in the draining lymph nodes of COVID-19, except for two control cases clustering together with the COVID-19 cases (a case of non-SARS related DAD and a case of pneumococcus pneumonia) (**Figure 7**). Compared to control cases, and in line with above mentioned phenotypic differences (**Figures 1** **–** **3**) and the results of the pathway enrichment analysis (**Figure 5**), there was a ***downregulation*** of multiple genes related to T-cell function and differentiation (*LCP2, CD3E, LEF1, CD6, MAL, TCL1A, TYROBP, CD84, CD5*), particularly related to CD4 cells (*IL16, CD4*), macrophage function (*LYZ, SIGLEC1*), genes encoding for immunoglobulin receptors (*FCER2*, *PIGR*) and transcription factors (*ETS1, EGR1, FOS*) in COVID-19. An ***upregulation*** of genes related to complement and fluid homeostasis and vascular functions (*SERPING1, AQP1, ADM, ANGPTL4*) was observed. Furthermore, there was evidence of aberrant leukocyte adhesion and motility (downregulation of *IRGB2*, upregulation of *ACTG2* and *DNASH12*). Genes encoding for enzymes involved in remodeling, such as *MMP9*, were downregulated (similarly shown in **Figure 4B**), while its inhibitor *TIMP1* as well as *ELN*, *PDGFRA* and *FN1* were upregulated. Expression patterns suggested a dysregulation in arachidonic acid metabolism: *PTGDS* (prostaglandin D2 synthase), was downregulated, while *CYP1B1* was upregulated.

## Discussion

To our knowledge, this is the first paper in the literature systematically characterizing COVID-19 lymph nodes in an effort to better understand the complex immunopathological changes of severe disease, both from a morphological and transcriptional level. Additionally, our dataset comprises COVID-19 patients from the first wave (March-July 2020) in Switzerland during which the use of high dose dexamethasone, which could have potentially served as a confounder, was limited (given to only 1/21 patients, presenting with clinical symptoms of HLH), thus potentially generating more robust evidence, and utilizes representative controls with a carefully selected spectrum of histological sequelae.

Our data demonstrates characteristic reaction patterns typical for severe COVID-19. Firstly, an unspecific albeit distinctive morphological response was observed, encompassing a three-fold accumulation of extrafollicular plasmablasts and a paucity and/or absence of germinal centers (**Figures 1B, C**). An accumulation of extrafollicular plasmablasts is a generally rarely observed, pathogenetically heterogeneous histological pattern most characteristically triggered by primary immune responses in mucosa-associated lymph nodes, occurring independently from germinal center formation and without establishment of an immunological memory (8). An accumulation of plasmablasts has previously been described in COVID-19 (5, 46–49). Its significance in this context is still unclear, although it may generate production of non- or sub-neutralizing antibodies which could result in antibody-mediated enhancement (AME), either by viral uptake into Fc-γ receptor IIa (FcγRIIa)-expressing phagocytic cells or by excessive antibody Fc-mediated immune complex formation. This would precipitate increased viral infection and replication in macrophages as well as an augmented inflammatory response, which could be one of the pathophysiological mechanisms contributing to severe COVID-19 (36). AME is a well-documented phenomenon in other viral diseases such as dengue fever and influenza (50, 51), although its role in SARS-CoV-2 immunology is still unknown. Potential evidence of AME in this study is demonstrated in **Figure 1F**, which features aggregates of SARS-CoV-2 N-protein in macrophages.

Secondly, several levels of evidence in this study suggest a dysregulation and/or exhaustion of T-cells. Six patients demonstrated a histologically evident reactivation of herpes viruses (EBV, HSV), which has also previously been described to have occurred in severe COVID-19 (52). T-cell dysfunction is known to play a critical role in herpes virus-family reactivation (53). Additionally, a significant paucity of FOXP3+, Tbet+ and LEF1+ lymphocytes in COVID-19 suggest a depletion of Treg and TH1 equivalents (**Figures 2A, B** and **3**); this is mirrored by the downregulation of key genes responsible for T-cell maturation and TCR signal transduction (**Figure 4B**, genes in green; **Figure 6B**) as well as *ICAM3*, *ITGB2* and *MMP9*, which are involved in leucocyte adhesion and migration. Pathway enrichment and clustering analysis similarly confirms these observations (**Figures 5**, **6B** and **7**). Taken together, these data imply a markedly deregulated and exhausted cellular immune response in severe COVID-19, particularly discernible in several T-cell compartments and checkpoints (54). It may also explain the high incidence of lymphopenia in severe COVID-19 (30, 55). Interestingly, neuropilin-1 which also serves as an entry factor for SARS-CoV-2, is highly expressed in mature FOXP3+ Tregs; this may imply viral tropism for Tregs, which may contribute to the T-cell dysregulation and decreased Treg equivalents observed here (56), warranting further study.

Thirdly, a pathological activation and phenotypic switch of macrophages, most notably reflected *in situ* by an increased hemophagocytic activity as well as clinically manifest HLH in one patient, was observed in a subset of our cohort (**Figure 1D**). As mentioned above and supported by previous evidence, HLH may be linked to the cytokine storm and aberrant antigen presentation in severe COVID-19 (12, 26). An increased fraction of a putative M2-polarized phenotype, as exemplified by an increase of CD163+ (and CD206+) cells in the lymph nodes, was similarly observed in lung samples of COVID-19 patients (57, 58). CD163+ macrophages, involved in the endocytotic uptake of free hemoglobin and haptoglobin, were previously shown to be increased in a broad spectrum of inflammatory disorders (59, 60). Soluble CD163+ (sCD163) is considered an effective biomarker of macrophage activation, which is frequently associated with hypercytokinemia (61). Accordingly, increased sCD163 was observed in COVID-19 patients (62), and a potential pathophysiological association between CD163+ macrophages and COVID-19-associated cytokine storm has recently been proposed by RNA-seq transcriptome analyses (57). Furthermore, an overall scarcity and downregulation of lysozyme, observed in the histological, gene expression and pathway enrichment data (**Figure 5**) of this study, could indicate an increased outflux and secretory activity of macrophages in the periphery (63). This would have to be confirmed by measuring the corresponding serum lysozyme (which would then be increased) (64), an issue requiring further investigation. Importantly, prior studies have shown that lysozyme can play a role in antiviral activity and suggest that low lysozyme levels (as found in COVID-19) may increase virulence (65, 66). *SIGLEC-1*, which negatively regulates viral infection-triggered type I IFN production (67) in macrophages, was downregulated in our dataset - this further underscores a macrophage-mediated dysregulated IFN-response as a hallmark of severe disease (68), as previously observed in lung tissue of the same autopsy cohort (17). In line with these findings, early clinical trials of otilimab, an anti-GM-CSF (granulocyte-macrophage colony stimulating factor) monoclonal antibody, showed a reduction of all-cause mortality in older patients with severe COVID-19 (69).

Finally, we present comprehensive evidence of widespread microvascular pathology affecting lymph nodes. An increased incidence of capillary stasis, accompanied by fibrin microthrombi (**Figure 1A**) was detected, findings consistent with systemic microthrombosis described in previous studies (12, 24, 70) and also our own on cardiac tissues (71) of the same cohort. Our findings imply that microangiopathy and thromboinflammation extends into lymphatic tissues, and its extent may be also linked to outcome (in the case of CD105, see **Supplementary Figure 1**). These histomorphological observations are further supported by GEP (**Figure 4B**, genes marked in red), which features a significant upregulation of genes essential for hemostasis (*CD36, PROCR, VWF*) and angiogenesis (*FLT1, TEK*), and in gene set enrichment and clustering analyses (**Figure 6A**). These findings engender likely therapeutic targets in severe COVID-19-associated thrombotic disease such as caplacizumab, an anti-vWF humanized immunoglobulin, which is currently approved for the use in thrombotic thrombocytopenic purpura (TTP) (72). Additionally, evidence of an upregulation of complement-associated genes (**Supplementary Figure 3**) imply a potential therapeutic use of C5a receptor inhibitors such as eculizumab or avacopan in severe disease (73, 74).

A dysregulation of arachidonic acid metabolism was observed in this cohort, evident in the upregulation of *CYP1B1* and the downregulation of *PTDGS (* **Figure 7** *)*. PTDGS catalyzes the conversion of prostaglandin H2 (PGH2) to prostaglandin D2, while *CYP1B1* converts arachidonic acid to epoxyeicosatrienoic acid regioisomers [EpETrE; functioning as lipid mediators in the vascular system (75)]. This raises questions regarding pharmacological agents, such as COX2 inhibitors, in the treatment of COVID-19, especially due to initial reports demonstrating activated COX2 expression by the SARS-CoV N-protein (76). This subsequently led to investigations on COX2 inhibitors such as celecoxib in SARS and COVID-19 with some promising results (77). Notably, the downregulation/knock-out of *PTGDS* is known to increase vascular permeability, neutrophilic influx and lung damage, in line with the clinical presentation of severe COVID-19 (78), which might be counteracted by COX2 inhibition (79). Thus, the therapeutic significance of pharmaceutic agents interfering with arachidonic acid metabolism (68) requires further investigation in adequately powered, randomized controlled trials.

This study has several limitations. Its retrospective design may not allow sufficiently accurate measurement of potential confounders, which may have influenced GEP or histological data. Furthermore, a sample size of 20 patients may not adequately capture the heterogeneity of COVID-19 immunology; thus, results such as the incidence of herpes-virus-family reactivation in the COVID-19 subgroup should be interpreted with caution. Future studies may provide further insight by comparing lymph nodes with peripheral blood, and other lymphatic organs such as bone marrow, spleen and tonsils, and also making use of single cell sequencing to unravel the subtle expression differences between different leucocyte populations at various sites.

In summary, the immunopathological effects of SARS-CoV-2 are heterogeneous, affecting both innate and adoptive pathways. Thromboinflammatory effects of COVID-19 extend to the lymphatic tissue, highlighting the central role of vascular dysfunction in severe disease. The data in this study thus underscore that severe/lethal COVID-19 is a predominantly angiocentric disease with profound immune dysregulation, possibly without the formation of an immunological memory.

## Data Availability Statement

The datasets presented in this study can be found in online repositories. The names of the repository/repositories and accession number(s) can be found below: https://www.ncbi.nlm.nih.gov/geo/, GSE189199.

## Ethics Statement

The studies involving human participants were reviewed and approved by Ethics Committee of Northwestern and Central Switzerland. The patients/participants provided their written informed consent to participate in this study. Written informed consent was obtained from the individual(s) for the publication of any potentially identifiable images or data included in this article.

## Author Contributions

JH, AT, and MM designed the study. JH and AT compiled the manuscript. Statistical analysis by CZ. Clinical data acquisition by SB, AS, JH, PW, and KM. RT-PCR and GEP assays by JS. Histomorphology and immunohistochemistry scoring by MM, JH, AT, and TM. Critical revision of the manuscript by KM, PW, SB, MM, CZ, AS, and JS. All authors contributed to the article and approved the submitted version.

## Funding

This study was funded by Botnar Research Centre for Child Health (BRCCH), Grant Nr: FTC-2020-10.

## Conflict of Interest

The authors declare that the research was conducted in the absence of any commercial or financial relationships that could be construed as a potential conflict of interest.

## Publisher’s Note

All claims expressed in this article are solely those of the authors and do not necessarily represent those of their affiliated organizations, or those of the publisher, the editors and the reviewers. Any product that may be evaluated in this article, or claim that may be made by its manufacturer, is not guaranteed or endorsed by the publisher.
