# Supplementary material for: Vascular Damage, Thromboinflammation, Plasmablast Activation, T-Cell Dysregulation and Pathological Histiocytic Response in Pulmonary Draining Lymph Nodes of COVID-19
Source: Front Immunol. 2021 Dec 13;12:763098. doi: 10.3389/fimmu.2021.763098 (PMC8710573; doi:10.3389/fimmu.2021.763098)
Supplement: Supplementary file 1 [file DataSheet_1.docx]

Supplementary Material

# Supplementary Methods

## Statistical analysis of immunohistochemical markers

Statistics was performed with IBM® SPSS®, Version 25 (Armonk, NY, USA). For categorical data, 2-sided Fisher’s exact-tests was applied. For non-parametric variables, a Mann-Whitney U-test was used, while parametric variables were computed with Student’s t-test. Correlation analyses were calculated with Spearman ρ. p-Values below 0.05 were considered significant. No adjustment for multiple testing was applied as the setting was considered hypothesis generating/exploratory.

## Antibody Protocol and Scoring Algorithms

*Supplementary Table 1*

| **Marker** | **Antibody source & clone** | **Retrieval**  **Dilution**  **Incubation** | **Scoring** |
| --- | --- | --- | --- |
| *Leukocyte/Vascular/Immune Markers* | | | |
| ACE2 | Abcam ab15348  Rabbit polyclonal | CC1 64 min  1:10000  12 min | Qualitative, descriptive assessment of lymphocytes; negative in granulocytes; perinodal fat as internal control |
| CD3 | Ventana 790-4341  Clone 2GV6 | CC1 32 min  Ready-to-use (RTU)  4 min | % positively staining T-cells in the paracortical (T-cell-) zones |
| CD4 | Ventana 790-4423  Clone SP35 | CC1 24 min  RTU  4 min | % positively staining T-cells in the paracortical (T-cell-) zones; weakly staining larger histiocytes/macrophages excluded |
| CD5 | Ventana 790-4451  Clone SP19 | CC1 32 min  RTU  12 min | % positively staining T-cells in the paracortical (T-cell-) zones |
| CD8 | Ventana 790-4460  Clone SP57 | CC1 24 min  RTU  16 min | % positively staining T-cells in the paracortical (T-cell-) zones |
| CD11c | Cellmarque 111M-18  Clone 5D11 | CC1 32 min  RTU  24 min | % positively staining histiocytes/ macrophages in the paracortical zones and in the sinus, respectively |
| CD14 | Ventana 760-4523  Clone EPR3653 | CC1 24 min  RTU  20 min | % positively staining histiocytes/macrophages in the paracortical zones and in the sinus, respectively |
| CD16 | Invitrogen PA5-80622  Rabbit polyclonal | CC1 16 min  1:400  20 min | % positively staining larger histiocytes/macrophages in the paracortical zones and in the sinus, respectively, and smaller intensively staining NK-cells |
| CD20 | Ventana 760-2531  Clone L26 | CC1 32 min  RTU  8 min | Qualitative, descriptive positivity in B-cells/cortical (B-cell-) zones |
| CD21 | Ventana 760-4245  Clone 2G9 | CC1 32 min  RTU  20 min | 0=physiological  1= disrupted FDC networks*  Disruption is defined by a pathological loss of the networks formed by follicular dendritic cells in germinal centres. |
| CD25 | Ventana 760-4439  Clone 4C9 | CC1 16 min  RTU  20 min | % positively staining T-cells in the paracortical (T-cell-) zones |
| CD68 | Dako IR613  Clone PG-M1 | CC1 32 min  RTU  4 min | % positively staining histiocytes/macrophages in the paracortical zones and in the sinus, respectively |
| CD105 | Abcam ab170943  Clone EPR10145-10 | CC1 16 min  1:100  20 min | Number of positively stained intranodal vessels per core (1.33mm²) |
| CD123 | Leica NCL-L-CD123  Clone BR4MS | CC1 40 min  1:20  32 min | % positively staining PDC cells in the paracortical (T-cell-) zones |
| CD163* | Ventana 760-4437  Clone MRQ-26 | CC1 32 min  RTU  20 min | % positively staining histiocytes/macrophages in the paracortical zones and in the sinus, respectively (M2 polarized phenotype) |
| CD206 | Cell Signaling cs-91992  Clone E2L9N9 | CC1 16 min  1:100  20 min | % positively staining histiocytes/macrophages in the paracortical zones and in the sinus, respectively (M2 polarized phenotype) |
| CXCL13 | Abcam ab112521  Rabbit polyclonal | CC1 48 min  1:100  24 min | % positively staining IDC in the paracortical zones |
| Fibrin | Dako A0080  Rabbit polyclonal | Pronase 30 min  1:100000  overnight | 0=negative  1=physiological, weak positivity of endothelial wall  2=moderate formation of fibrin mesh  3=fibrin microthrombi |
| FOXP3 | Abcam ab99963  Clone SP97 | CC1 40 min  1:50  32 min | % positively staining regulatory T-cells |
| F VIII-R (vWF) | Ventana 760-2642  Rabbit polyclonal | CC1 24 min  RTU  20 min | 0=no or only single (<5%) positive cells (histiocytes/macrophages) in the paracortical zones  1=≥5% positive histiocytes/ macrophages in the paracortical zones |
| F XIIIA | Ventana 760-4441  Clone EP3372 | CC1 32 min  RTU  12 min | % positively staining histiocytes/macrophages in the paracortical zones and in the sinus, respectively |
| GATA3 | Ventana 760-4897  Clone L50-823 | CC1 32 min  RTU  16 min | % positively staining TH2-cells in the paracortical (T-cell-) zones |
| HLA-DR | Dako M0775  Clone CR/43 | CC1 32 min  1:50  20 min | % positively staining rather M1 polarized histiocytes/macrophages in the paracortical zones and in the sinus, respectively |
| IgA | Ventana 760-2652  Rabbit polyclonal | CC1 8 min  RTU  12 min | % positively staining plasma cells |
| IgD | Ventana 760-4444  Rabbit polyclonal | CC1 16 min  RTU  20 min | % positively staining plasma cells |
| IgG | Ventana 760-2653  Rabbit polyclonal | CC1 8 min  RTU  12 min | % positively staining plasma cells |
| IgG4 | Ventana 760-4614  Clone MRQ-44 | CC1 16 min  RTU  16 min | % positively staining plasma cells |
| IgM | Ventana 760-2654  Rabbit poly | CC1 8 min  RTU  12 min | % positively staining plasma cells |
| LEF1 | Abcam ab137872  Clone EPR2029Y | CC1 32 min  1:50  32 min | % intensively positive staining T-cells in the paracortical (T-cell-) zones |
| Lysozyme* | Ventana 760-2656  Rabbit polyclonal | CC1 8 min  RTU  16 min | % positively staining histiocytes/macrophages in the paracortical zones and in the sinus, respectively (M1 polarized phenotype) |
| MUM1p (IRF4) | Ventana 760-4529  Clone MRQ-43 | CC1 32 min  RTU  24 min | % positively staining plasmablasts excluding plasma cells (which are smaller than plasmablasts) |
| NKG2A | Abcam ab260035  Clone EPR23737-127 | CC1 16 min  1:200  20 min | % positively staining NK-cells |
| PD1 | Ventana 760-4895  Clone NAT105 | CC1 48 min  RTU  12 min | % weakly positive staining (exhausted) T-cells in the paracortical (T-cell-) zones |
| PDL1 | Ventana 790-4905  Clone SP263 | CC1 56 min  RTU  32 min | % positively staining histiocytes/ macrophages in the paracortical zones and in the sinus (enclosing sinus-lining cells), respectively |
| RORγ | Biocare Medical API328 AA  Clone 6F3.1 | Mikrowelle EDTA 98°C 30 min  RTU | % positively staining TH17-cells in the paracortical (T-cell-) zones |
| T-Bet | Abcam ab154200  Clone EPR9302 | CC1 16  1:100  32 min | % positively staining TH1-cells in the paracortical (T-cell-) zones |
| TCRβF1 | Thermo TCR1151  Clone 8A3 | Protease1 4 min  1:50  40 min | % positively staining αβ T-cells in the paracortical (T-cell-) zones |
| TCRδ | Santa Cruz sc-100289  Clone H41 | CC1 48 min  1:50  60 min | % positively staining γδ T-cells in the paracortical (T-cell-) zones |
| VEGF | Thermo MA5-13182  Clone JH121 | CC1 16 min  1:50  24 min | 1=weak expression in (a few) paracortical cells  2=moderate expression in (a substantial proportion) of paracortical cells  3=strong expression in (almost all) paracortical cells |
| *Viruses* | | | |
| Adenovirus | Ventana 760-4870  Clone 2/6+20/11 | Protease1 8 min  RTU  8 min | Any specific (nuclear) positivity |
| CMV | Dako IR752  Clone CCH2+DDG9 | Protease1 4 min  RTU  12 min | Any specific (nuclear) positivity |
| EBER ISH | Ventana 800-2842  RNA Probe | ISH Protease3 4 min  RTU  6h | Any nuclear positivity  1-9%: suggestive of previous infection, inactive, considered negative  ≥10%: suggestive of loss of immunologic control over EBV infection, considered positive |
| HHV8 | Ventana 760-4260  Clone 13B10 | CC1 24 min  RTU  20 min | Any specific (nuclear) positivity |
| HSV | Ventana 760-4350  Clone OCH1E5 | CC1 32 min  RTU  24 min | Any specific (nuclear) positivity |
| Parvovirus B19 | Cellmarque 218M-16  Clone R92F6 | CC1 24 min  1:100  32 min | Any specific (nuclear) positivity |
| SARS-CoV-2 ISH | As described in: Reinhold A et al. Ocular pathology and occasionally detectable intraocular SARS-CoV-2 RNA in five fatal COVID-19 cases. Ophthalmic Res. 2021 Jan 20. doi: 10.1159/000514573. | | Granular cytoplasmic positivity with staining intensity as strong as in RT-qPCR positive infected lungs with high virus load |
| SARS-CoV-2 N-antigen | Rockland 200-401-A50  Rabbit poly | CC1 36 min  1:2000  24 min | Granular cytoplasmic positivity with staining intensity as strong as in RT-qPCR positive infected lungs with high virus load |
| SV40 Polyomavirus | Ventana 760-4449  Clone MRQ4 | CC1 32 min  RTU  8 min | Any specific (nuclear) positivity |
| VZV | Cellarque 364M-18  Clone SG1-1+SG1-SG4+NCP-1+IE62 | CC1 32 min  RTU  8 min | Any specific (nuclear) positivity |

* M1/M2 ratio = % positively staining lysozyme / % positively staining CD163 histiocytes

# Supplementary Results

## CD105+ Micorovessel Density

**
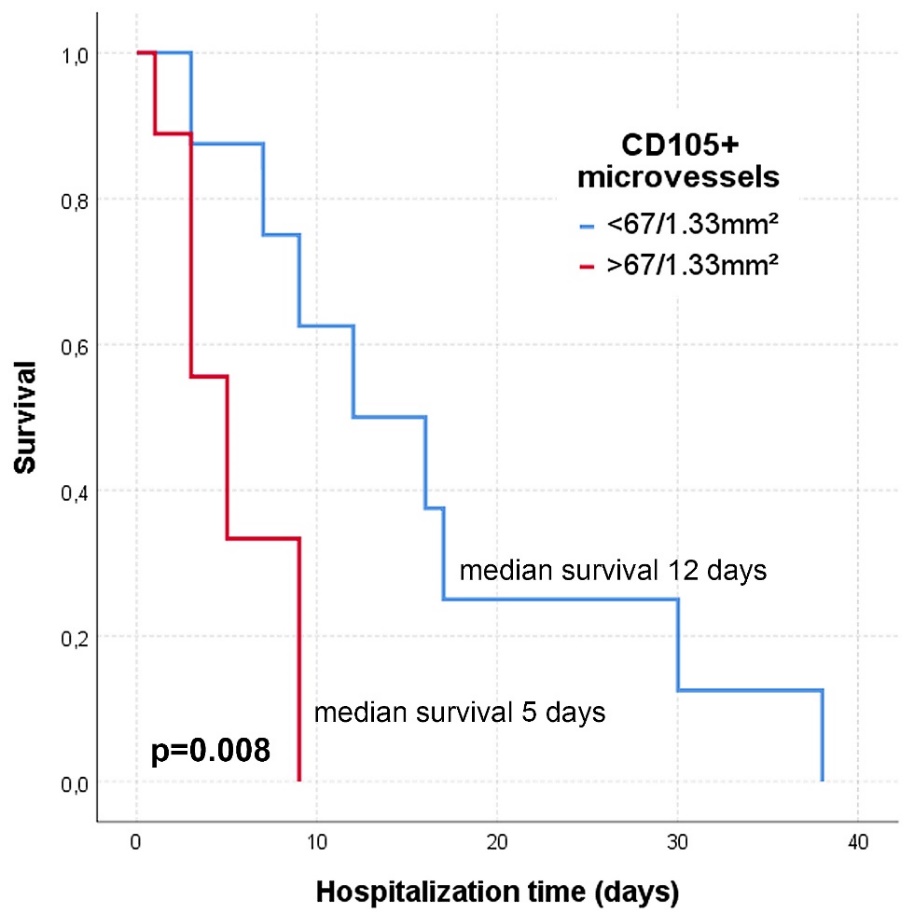
**

**Supplementary Figure 1.** Kaplan-Meier survival curves demonstrating a significant impact on survival length, dependent on density of CD105+ vessels in COVID-19 patients; the cut-off score of 67 CD105+ microvessels has been calculated applying ROC-curve analysis as described (Tzankov A et al. Prognostic immunophenotypic biomarker studies in diffuse large B cell lymphoma with special emphasis on rational determination of cut-off scores. Leuk Lymphoma. 2010;51:199-212).

## Clinical, Serological and Histological Characteristics – Correlation Analysis

**COVID-19**

**Controls**

BMI: body mass index; CRP: C-reactive protein; CS: capillary stasis; FIB: fibrin microthrombi; GC: germinal centers; HLH: hemophagocytic lymphohistiocytosis; HT: hospitalization time; IL6: interleukin 6; LDH: Lactate dehydrogenase; LEU: leucocytes 10^9^/L; LYM: lymphocytes 10^9^/L; MON: monocytes 10^9^/L; NEU: Neutrophilic granulocytes 10^-9/l; PB: plasmablasts; PC: plasma cells; ThT: thrombin time; VL: viral load

**Supplementary Figure 2:** Spearman Rho correlation analysis of clinical, serological and histological characteristics of COVID-19 vs. controls shows markedly different correlational relationships in individual groups. Correlations with 0.05 level significance denoted in bold. Correlations with 0.01 level significance denoted in bold and underlined.

## Complement-related Genes


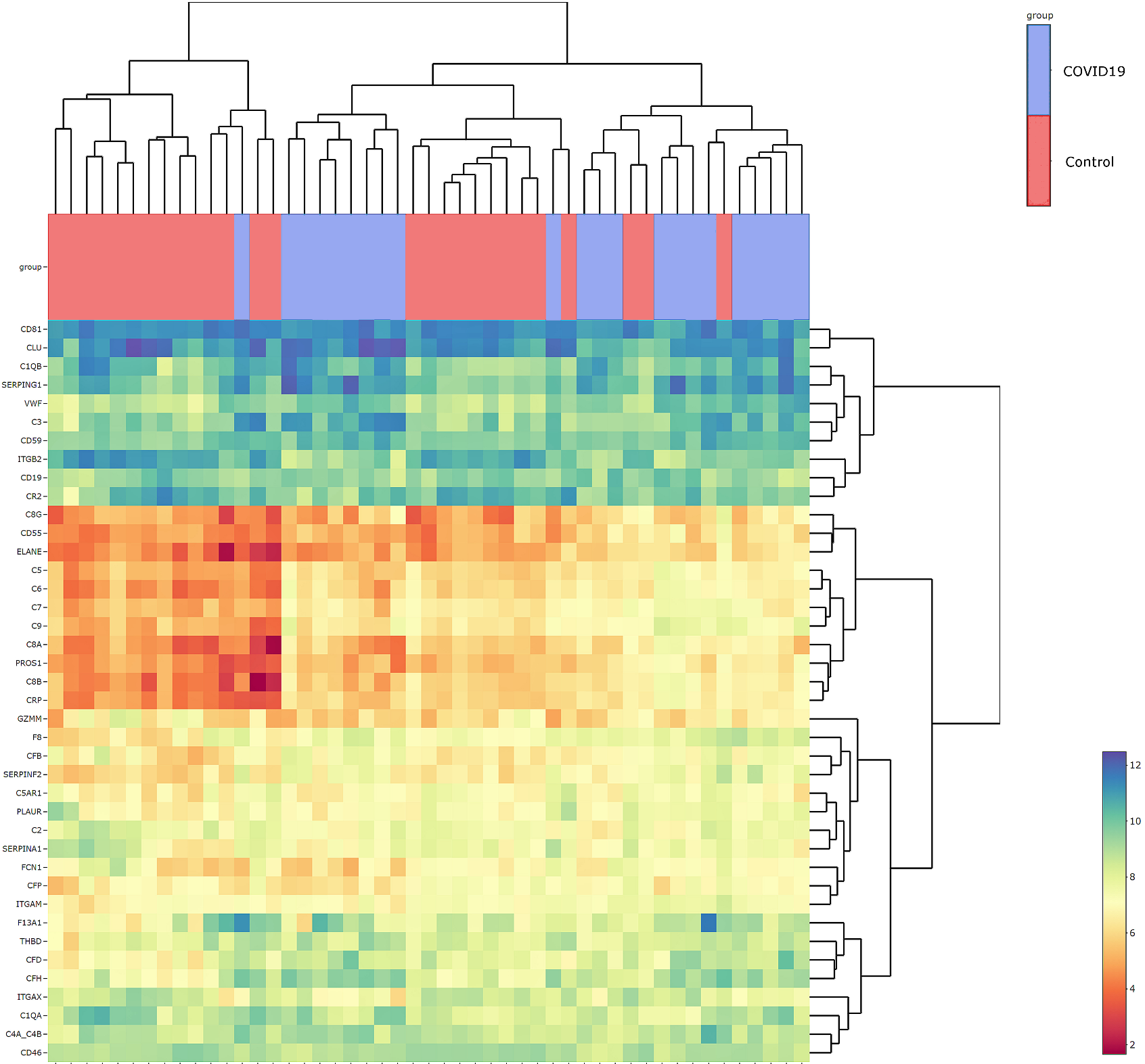


CD: cluster of differentiation; CLU: clusterin, C1QB: Complement C1q B Chain; SERPING1: Serpin Family G Member 1; VWF: von Willebrand Factor; C: complement; ITGB2: Integrin Subunit Beta 2; CR2: Complement C3d Receptor 2; C8G: Complement C8 Gamma Chain; ELANE: Elastase, Neutrophil Expressed; C8A: Complement C8 Alpha Chain; PROS1: Protein S; C8B: Complement C8 Beta Chain; CRP: C-reactive protein; GZMM: Granzyme M; F8: factor VIII; CFB: Complement Factor B; SERPINF2: Serpin Family F Member 2; C5AR1: Complement C5a Receptor 1; PLAUR: Plasminogen Activator, Urokinase Receptor; SERPINA1: Serpin Family A Member 1; FCN1: Ficolin 1; CFP: Complement Factor Properdin; ITGAM: Integrin Subunit Alpha M; F13A1: Coagulation Factor XIII A Chain; THBD: Thrombomodulin; CFD: Complement Factor D; CFH: Complement Factor H; ITGAX: Integrin Subunit Alpha X; C1QA: Complement C1q A Chain

**Supplementary Figure 3:** Clustering analysis of complement-related gene set (according to the HTG EdgeSeq Reveal algorithms). A relative overexpression of complement-related, e.g. *C1QB* and *C3*, and prothrombotic genes, e.g. *SERPING1* and *VWF* is observedin COVID-19.

## *
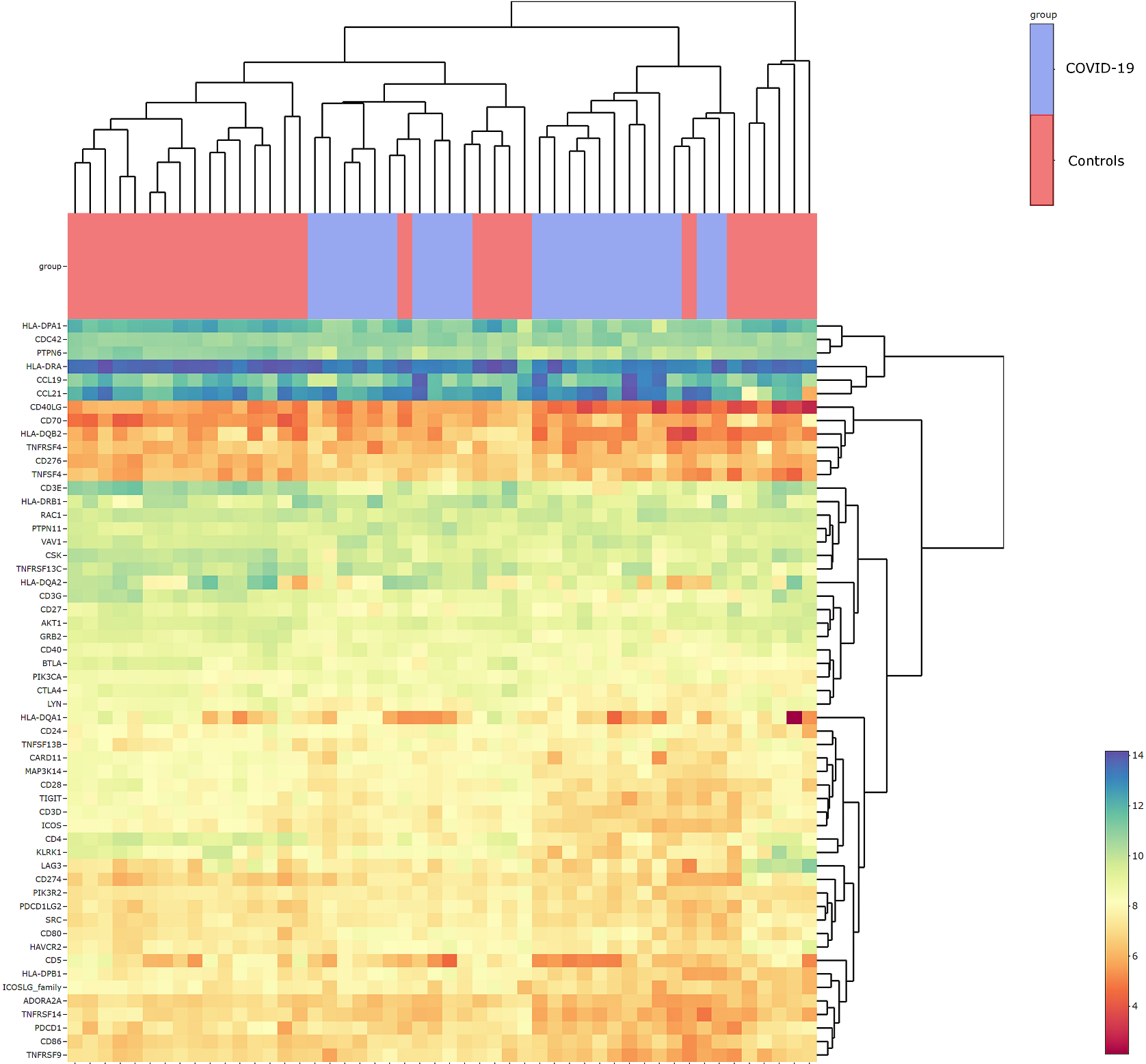
*T-Cell Checkpoint-related Genes

HLA-DPA1: Major Histocompatibility Complex, Class II, DP Alpha 1; CDC42: Cell Division Cycle 42; PTPN6: Protein Tyrosine Phosphatase Non-Receptor Type 6; CCL: Chemokine (C–C motif) ligand; CD: cluster of differentiation; CD40LG: cluster of differentiation 40 ligand; HLA-DQB2: Major Histocompatibility Complex, Class II, DQ Beta 2; TNF(R)SF: TNF (Receptor) Superfamily Member; HLA-DRB1: Major Histocompatibility Complex, Class II, DR Beta 1; RAC1: Rac Family Small GTPase 1; PTPN11: Protein Tyrosine Phosphatase Non-Receptor Type 11; VAV1: Vav Guanine Nucleotide Exchange Factor 1; CSK: C-Terminal Src Kinase; AKT1: AKT Serine/Threonine Kinase 1; GRB2: Growth Factor Receptor Bound Protein 2; BTLA: B And T Lymphocyte Associated; PIK3CA: Phosphatidylinositol-4,5-Bisphosphate 3-Kinase Catalytic Subunit Alpha; CTLA4: Cytotoxic T-Lymphocyte Associated Protein 4; LYN: LYN Proto-Oncogene, Src Family Tyrosine Kinase; HLA-DQA1: Major Histocompatibility Complex, Class II, DQ Alpha 1; CARD11: Caspase Recruitment Domain Family Member 11; MAP3K14: MAP 3 Kinase 14; TIGIT: T cell immunoreceptor with Ig and ITIM domains; ICOS (LG): Inducible T Cell Costimulator (ligand); KLRK1: Killer Cell Lectin Like Receptor K1; LAG3: Lymphocyte Activating 3; PIK3R2: Phosphoinositide-3-Kinase Regulatory Subunit 2; PDCD1LG2: Programmed Cell Death 1 Ligand 2; SRC: SRC Proto-Oncogene; HAVCR2: Hepatitis A Virus Cellular Receptor 2; HLA-DPB1: Major Histocompatibility Complex, Class II, DP Beta 1; ADORA2A: Adenosine A2a Receptor; PDCD1: Programmed cell death protein 1

**Supplementary Figure 4:** Clustering analysis of T-cell checkpoint-related gene set (according to the HTG EdgeSeq Reveal algorithms). A general relative downregulation of T-cell function-related genes, e.g. *CD3E* and *CD27* is observed in COVID-19.

# List of p-Values of Angiogenesis Related Genes (Addendum to Figure 6A)

*Supplementary Table 2*

| **Probe** | **Fold Change COVID.vs.Control** | **rawP COVID.vs.Control** | **adjP COVID.vs.Control** |
| --- | --- | --- | --- |
| **F2** | 1.26 | 0.175 | 0.232 |
| **ANG** | **1.52** | **6.40 x 10^-3^ **** | **1.28 x 10^-2^ **** |
| **ANGPT2** | **1.61** | **3.70 x 10^-4^ **** | **1.10 x 10^-3^ **** |
| **AGT** | **2.03** | **3.60 x 10^-8^ **** | **3.24 x 10^-7^ **** |
| **ANGPTL1** | **2.05** | **1.49 x 10^-7^ **** | **1.17 x 10^-6^ **** |
| **ANGPT1** | **1.99** | **3.41 x 10^-9^ **** | **4.19 x 10^-8^ **** |
| **BMPER** | **1.68** | **2.45 x 10^-4^ **** | **7.34 x 10^-4^ **** |
| **FLT3** | -1.21 | 7.46 x 10^-2^ | 1.11 x 10^-1^ |
| **FOXP1** | **-1.63** | **1.22 x 10^-6^ **** | **7.04 x 10^-6^ **** |
| **FLT3LG** | **-1.55** | **6.84 x 10^-5^ **** | **2.39 x 10^-4^ **** |
| **SMAD4** | -1.13 | 3.79 x 10^-2^ * | 6.11 x 10^-2^ |
| **TGFBR1** | **-1.52** | **5.15 x 10^-13^ **** | **2.15 x 10^-11^ **** |
| **P2RX4** | 1.05 | 0.318 | 0.383 |
| **PRMT5** | **1.22** | **8.50 x 10^-3^ **** | **1.64 x 10^-2^ **** |
| **SOCS5** | **1.27** | **1.00 x 10^-3^ **** | **2.50 x 10^-3^ **** |
| **TIMP2** | 1.19 | 5.25x 10^-2^ * | 8.10 x 10^-2^ |
| **CAV1** | **2.25** | **1.76 x 10^-11^ **** | **4.10 x 10^-10^ **** |
| **CAVIN2** | **1.86** | **9.64 x 10^-5^ **** | **3.22 x 10^-4^ **** |
| **CXCL10** | **-2.69** | **3.22 x 10^-4^ **** | **9.38 x 10^-4^ **** |
| **SERPINF1** | **1.89** | **3.04 x 10^-7^ **** | **2.10 x 10^-6^ **** |
| **PECAM1** | **1.25** | **2.81 x 10^-2^ *** | **4.68 x 10^-2^ *** |
| **HSH2D** | 1.04 | 0.706 | 0.754 |
| **TCF4** | 1.00 | 0.997 | 0.998 |

# List of GSEA, KEGG and Gene Ontology Datasets

*Supplementary Table 3*

| **Name in Figure** | **Pathway Name** | **Link** |
| --- | --- | --- |
| **E2F Targets** | HALLMARK_E2F_TARGETS | <https://www.gsea-msigdb.org/gsea/msigdb/cards/HALLMARK_E2F_TARGETS> |
| **Influenza Vaccine Response** | GSE29614_CTRL_VS_DAY7_TIV_FLU_VACCINE_PBMC_DN | <https://www.gsea-msigdb.org/gsea/msigdb/cards/GSE29614_CTRL_VS_DAY7_TIV_FLU_VACCINE_PBMC_DN.html> |
| **Memory CD4 T-Cell Response upon Stimulation** | GSE36476_CTRL_VS_TSST_ACT_72H_MEMORY_CD4_TCELL_OLD_DN | <https://www.gsea-msigdb.org/gsea/msigdb/cards/GSE36476_CTRL_VS_TSST_ACT_72H_MEMORY_CD4_TCELL_OLD_DN.html> |
| **Chromosome Segregation** | GO_chromosome_segregation | <http://amigo.geneontology.org/amigo/term/GO:0007059> |
| **Nuclear Division** | GO_mitotic_nuclear_division (GO:0140014) | <http://amigo.geneontology.org/amigo/term/GO:0140014> |
| **Chromatid Segregation** | GO_sister_chromatid_segregation (GO:0000819) | <http://amigo.geneontology.org/amigo/term/GO:0000819> |
| **G2M Checkpoint** | HALLMARK_G2M_CHECKPOINT | <https://www.gsea-msigdb.org/gsea/msigdb/cards/HALLMARK_G2M_CHECKPOINT.html> |
| **Rename to “Metaphase-Anaphase Transition”**  **(Gorilla – OK)** | GO_metaphase_anaphase_transition_of_cell_cycle (GO:0007091) | <http://amigo.geneontology.org/amigo/term/GO:0007091> |
| **Chromosome Separation** | GO_regulation_of_chromosome_separation (GO:1905818) | <http://amigo.geneontology.org/amigo/term/GO:1905818> |
| **MYC Targets** | HALLMARK_MYC_TARGETS_V1 | <https://www.gsea-msigdb.org/gsea/msigdb/cards/HALLMARK_MYC_TARGETS_V1.html> |
| **Extracellular Matrix Receptor Interaction** | KEGG_ECM_RECEPTOR_INTERACTION | <https://www.gsea-msigdb.org/gsea/msigdb/cards/KEGG_ECM_RECEPTOR_INTERACTION> |
| **MTORC1 Signaling** | HALLMARK_MTORC1_SIGNALING | <https://www.gsea-msigdb.org/gsea/msigdb/cards/HALLMARK_MTORC1_SIGNALING.html> |
| **Cell Cycle** | KEGG_CELL_CYCLE | <https://www.gsea-msigdb.org/gsea/msigdb/cards/KEGG_CELL_CYCLE.html> |
| **Epithelial-Mesenchymal Transition** | HALLMARK_EPITHELIAL_MESENCHYMAL_TRANSITION | <https://www.gsea-msigdb.org/gsea/msigdb/cards/HALLMARK_EPITHELIAL_MESENCHYMAL_TRANSITION.html> |
| **Fatty Acid Metabolism** | HALLMARK_FATTY_ACID_METABOLISM | <https://www.gsea-msigdb.org/gsea/msigdb/cards/HALLMARK_FATTY_ACID_METABOLISM.html> |
| **Hematopoietic Lineage Development** | KEGG_HEMATOPOIETIC_CELL_LINEAGE | <https://www.gsea-msigdb.org/gsea/msigdb/cards/KEGG_HEMATOPOIETIC_CELL_LINEAGE.html> |
| **B-Cell Receptor Signaling** | KEGG_B_CELL_RECEPTOR_SIGNALING_PATHWAY | <https://www.gsea-msigdb.org/gsea/msigdb/cards/KEGG_B_CELL_RECEPTOR_SIGNALING_PATHWAY.html> |
| **Viral Myocarditis** | KEGG_VIRAL_MYOCARDITIS | <https://www.gsea-msigdb.org/gsea/msigdb/cards/KEGG_VIRAL_MYOCARDITIS.html> |
| **Lysozyme Function** | KEGG_LYSOSOME | <https://www.gsea-msigdb.org/gsea/msigdb/cards/KEGG_LYSOSOME.html> |
| **Graft Versus Host Disease** | KEGG_GRAFT_VERSUS_HOST_DISEASE | <https://www.gsea-msigdb.org/gsea/msigdb/cards/KEGG_GRAFT_VERSUS_HOST_DISEASE.html> |
| **Intercellular Adhesion Molecules** | KEGG_CELL_ADHESION_MOLECULES_CAMS | <https://www.gsea-msigdb.org/gsea/msigdb/cards/KEGG_CELL_ADHESION_MOLECULES_CAMS.html> |
| **Immunopathology of Diabetes Mellitus Type I** | KEGG_TYPE_I_DIABETES_MELLITUS | <https://www.gsea-msigdb.org/gsea/msigdb/cards/KEGG_TYPE_I_DIABETES_MELLITUS.html> |
| **TNFα Signaling via NF-κB** | HALLMARK_TNFA_SIGNALING_VIA_NFKB | <https://www.gsea-msigdb.org/gsea/msigdb/cards/HALLMARK_TNFA_SIGNALING_VIA_NFKB.html> |
| **Allograft Rejection** | HALLMARK_ALLOGRAFT_REJECTION | <https://www.gsea-msigdb.org/gsea/msigdb/cards/HALLMARK_ALLOGRAFT_REJECTION.html> |
